# Characterization of Flexusin A, a Novel Circular Bacteriocin Produced by Marine Bacterium *Bacillus flexus* R29-2

**DOI:** 10.3390/md23030095

**Published:** 2025-02-21

**Authors:** Xiaoni Qiao, Xiaowen Sun, Shuting Wang, Chen Zhai, Wei Tang, Tao Tang, Jun Zhang, Zengguo He

**Affiliations:** 1 School of Medicine and Pharmacy, Ocean University of China, Qingdao 266003, China; qiaoxiaoni@stu.ouc.edu.cn (X.Q.); sxw1117@126.com (X.S.); shuting0302@163.com (S.W.); tianti_121@163.com (W.T.); 2016306@ouc.edu.cn (T.T.); zhangjun841013@aliyun.com (J.Z.); 2 Qingdao Bioantai Biotechnology Co., Ltd., Qingdao 266000, China; 3 State Key Laboratory of Animal Nutrition, Institute of Animal Sciences of Chinese Academy of Agricultural Sciences, Beijing 100193, China; zhaichen@caas.cn; 4 Marine Microbial Engineering Research and Development Center, Marine Biomedical Research Institute of Qingdao, Qingdao 266071, China

**Keywords:** *Bacillus flexus* R29-2, circular bacteriocin, characterization, leader peptide, mechanism of action

## Abstract

Circular bacteriocins are potent antimicrobials against pathogenic Gram-positives. In searching for marine bacteriocins, an antibacterial peptide (flexusin A) was purified from the fermentation broth of marine bacterium *Bacillus flexus* R29-2. Genome sequencing and gene annotation revealed the chromosome contained an unknown circular bacteriocin gene cluster. Approaches including shot-gun proteomics analysis, AntiSMASH and BAGEL4 predication as well as the comprehensive sequence alignment, were then conducted, respectively, to verify the correlation of flexusin A with the gene-encoded precursor peptide. The results confirmed that flexusin A was the mature circular bacteriocin of the predicated precursor peptide with six amino acids as leader peptide. Flexusin A was 6098.4 Da in size, with a net charge of +3 and PI of 9.60. It shared the typical saposin-like fold spatial conformation features as commonly found in other circular bacteriocins. Flexusin A was pH, thermal, and protease tolerant. It exhibited a narrow antimicrobial spectrum against Gram-positives, and it can strongly inhibit *Staphylococcus aureus* by causing cell destruction via membrane destabilization. Taken together, a novel circular bacteriocin flexusin A was identified in this work. The characterization of flexusin A has extended circular bacteriocins family to 26 members. This is also the first report on bacteriocin production by *B. flexus*.

## 1. Introduction

The spread of drug resistance has posed as major challenge to global public health in the 21st century [1]. In 2017, World Health Organization published a priority list of pathogens called ESKAPE, the acronym of *Enterococcus faecium*, *Staphylococcus aureus*, *Klebsiella pneumoniae*, *Acinetobacter baumannii*, *Pseudomonas aeruginosa*, and *Enterobacter* spp., which are the common pathogenic bacteria associated with nosocomial infection [2]. Due to lack of new antibiotics with efficacy, the emerging drug-resistant “ESKAPE” have presented as a significant threat to the public health nowadays [3,4,5]. While limited antibiotics have been developed since the 1980s [6], the spreading and evolving of antibiotic-resistant “ESKAPE” bacteria have begun rendering most antimicrobials ineffective. The shortage of effective drugs for treating bacterial infections caused by the multidrug-resistant bacteria (superbugs) simply accelerated the advent of “post-antibiotic era” [5,7]. Unless new antibiotics and therapeutics are explored, the decimation of modern medicine will soon become a reality [7]. Demand to develop new antibacterials for controlling “ESKAPE” is on rising [2].

Bacteriocins are ribosome-synthesized antimicrobial peptides that inhibit other bacteria of similar taxonomic status [8,9]. In general, bacteriocins are relatively safe for mammals; some of them are facilitated with GRAS status [10], and just as exemplified by nisin, the well-known bacteriocin that has been widely used in food industry for nearly a century in Western countries [11]. Most of the well documented bacteriocins are active against Gram-positive bacteria, including the ones listed on “ESKAPE” [12,13,14,15,16]. So far, the risk of bacteriocins resistance for clinical use still remains unknown, since no clinical extensive usage has been approached and/or no resistance mechanisms have yet been characterized [17]. For membrane targeted bacteriocins, the resistances are not so prone to be established since structure-wise the target cell membrane is conserved and it would be lethal-orientated once the mutation occurs [18].

Circular bacteriocins are a class of bacteriocins with the N-terminal and C-terminal closed into a ring structure, which endows the bacteriocins with better stability such as pH, thermal, and protease tolerance [17]. To date, a panel of circular bacteriocins such as enterocin AS-48, carnocyclin A, enterocin NKR-5-3B, acidocin B and plantacyclin have already been fully elucidated [19]. The well-known mechanism of circular bacteriocins, e.g., the case for AS-48, is associated with the destabilization of target cell membrane through direct insertion into the lipid bilayer via electrostatic and hydrophobic interactions [20]. AS-48 was recently advancing into the preclinical characterization as reported, thanks to its featured activity against the antibiotic-resistant strains [21]. With new techs developed and applied, potentials of circular bacteriocins are continuously being uncovered [19,22,23,24,25,26].

Compared to terrestrial microorganisms, marine microorganisms represent a vast and largely unexplored reservoir of bioactive compounds with diverse industrial applications [27], of which some active compounds have been confirmed to be promising [28]. So far, around half of the reported marine-derived preclinical antimicrobial compounds identified were associated with microorganism. Among them marine-derived bacteriocins have attracted major interest from researchers [29,30,31,32].

In our recent screening for marine bacteriocinogenics, a deep-sea bacterium *Bacillus flexus* R29-2 was identified for its potential for producing bacteriocin-like substances. In the current study, whole genome sequencing of R29-2 was conducted to pinpoint the gene clusters relevant to the antimicrobials. The bacterium was submerged-fermented to obtain adequate active samples to facilitate downstream isolation and purification. Followed structural characterization via MALDI-MS and MS/MS together with the whole genome sequencing and bioinformatics analysis allowed the discovery of a novel circular bacteriocin with the designation of flexusin A. Lastly, the stability and the mechanism of action of the peptide was also sought.

## 2. Results and Discussion

### 2.1. Strain Screening and Identification

In the search for bacteriocinogenic bacteria a marine bacterial isolate R29-2 was identified for its narrow-spectrum inhibitory activity against Gram-positives including the ones listed in ESKAPE (Table 1). Through whole genome sequencing, the isolate R29-2 was identified as *Bacillus flexus*. The strain was deposited in the China General Microbiological Culture Collection Center (CGMCC) with accession number 18731.

### 2.2. Purification and Identification of Antibacterial Peptide Flexusin A

Spore formers, including the *Bacillus* species, are well known for antibacterial substance production [12,33]. Recently, we identified a new circular bacteriocin velezin together with 3 surfactin lipopeptides from the fermentate of *B. velezensis* 1-3 [34]. To illustrate the antimicrobials produced by isolate R29-2, a set of submerged fermentation techniques was then established at pilot-scale to obtain adequate quantity of antibacterial sample for further analysis.

**Figure 1:**
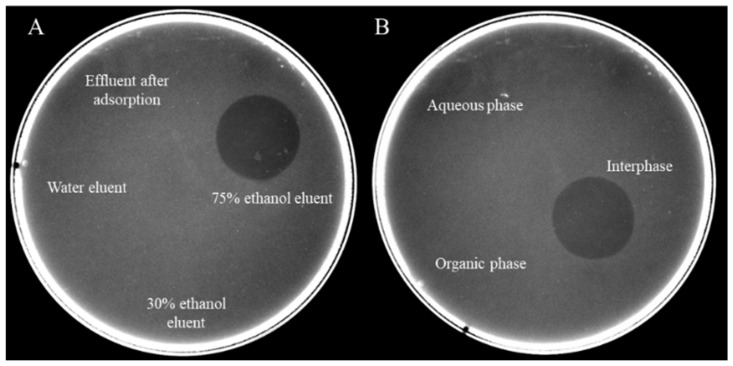
Antibacterial activity assay of (**A**) fractions during the macroporous resin purification process, and (**B**) the fractions during chloroform extraction.

**Figure 2:**
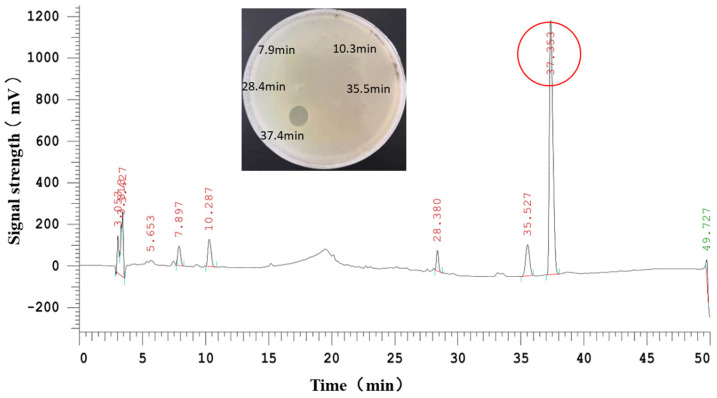
Purification of antibacterial bacteriocin-like substances by HPLC. The peaks with different retention times were collected for antibacterial activity assay, and the one showing activity was marked in red circle.

**Figure 3:**
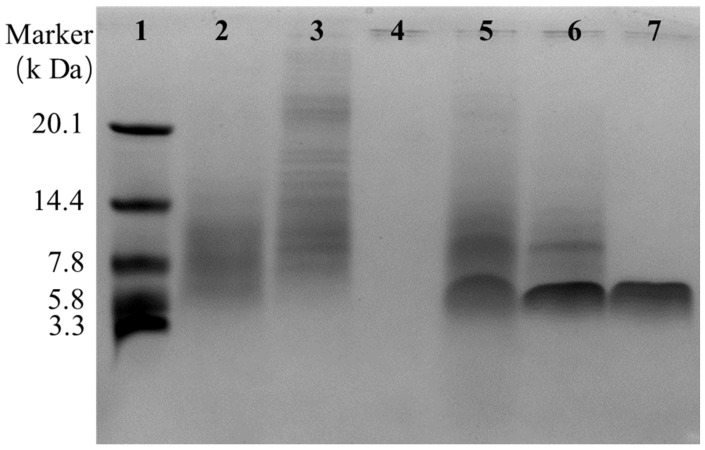
Tricine-SDS-PAGE of the fractions generated during isolation and purification. Lane 1, the marker; lane 2, cell-free fermentation supernatant; lane 3, water eluent; lane 4, 30% ethanol eluent; lane 5, 75% ethanol eluent; lane 6, interphase by chloroform extraction; lane 7, peak with antibacterial activity (retention time of 37.353 min) in HPLC profile.

It was found that the antibacterial substances of R29-2 could be isolated from the fermentation broth with one step macroporous resin adsorption manipulation as previously used elsewhere [12]. The antibacterials were further recovered from the 75% ethanol fraction after the impurities matters were removed through stepwise elution using distilled water and 30% ethanol sequentially (Figure 1A). A concentrated crude antibacterial sample was obtained by removing residual ethanol via rotary evaporation. The aqueous concentrate was then thoroughly mixed with trichloromethane followed by vigorously stirring, allowing the antibacterial substances to be enriched in the thin interface layer between the aqueous phase and the organic phase (Figure 1B). The antibacterial preparation was further subjected to reverse-phase HPLC for further purification. The antibacterial fraction (retention time of 37.353 min) was collected and lyophilized (Figure 2). The purified antibacterial preparation was then subjected to Tricine-SDS-PAGE analysis, from which one clear peptide band of ~6 kDa was identified (Figure 3). The antibacterial peptide was hereafter named as flexusin A.

**Figure 4:**
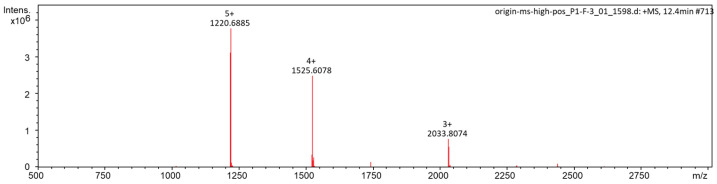
Detection of the molecular mass of flexusin A by UHPLC-QqQ MS.

The purified flexusin A was further subjected to UHPLC-QqQ MS for molecular mass determination (Figure 4). Three multiple-charged ions [M + 5H]^5+^ (*m*/*z* = 1220.6885), [M + 4H]^4+^ (*m*/*z* = 1525.6078) and [M + 3H]^3+^ (*m*/*z* = 2033.8074) were detected, and the molecular mass of flexusin A was calculated to be 6098.4 Da by the formula: M = m × n − n × 1.007, whereas M is the molecular mass of flexusin A, m is the mass of multi-charged ions detected and n is the charge number.

### 2.3. The Prediction of a Circular Bacteriocin Through Whole Genome Sequencing

**Figure 5:**
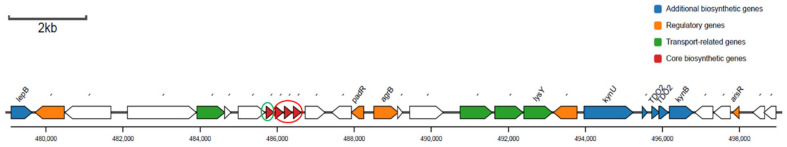
Prediction of gene cluster of circular bacteriocin BFCir-1.

Whole genome sequencing of *B. flexus* R29-2 allowed the identification of a chromosomal with 4,147,034 bp in length and five plasmid sequences. And all the sequences derived were uploaded to antiSMASH and BAGEL4 databases, respectively, for the metabolite genes prediction [35,36]. Interestingly, both antiSMASH and BAGEL4 databases did predict an unknown circular bacteriocin gene cluster located on the chromosome. Up to 31 genes were identified in the circular bacteriocin gene cluster, among them three duplicate structure gene copies (in red circle, termed as *bfcir-1* herein) were recognized (Figure 5). The structure gene encoded a precursor peptide (66 amino acids) with the sequence of MEVVAYIAGALGISEYWANVIVTAIEAGSTVLALISMFASFGLTSALILTAKSLLKKGAKKTAVAY. The precursor peptide had a calculated molecular mass of 6809.12 Da; it is termed as BFCir-1 hereafter. According to the database search, no known bacteriocin gene with high similarity to *bfcir-1* has been reported thus far [37].

Upstream closely adjacent to the three duplicate structure gene copies located a gene (marked with a green circle in Figure 5) has a high similarity to the structure gene, but the annotation failed to provide any clues regarding its functions. Several regulatory genes, including transport-related genes (*lys Y*, etc.), and signaling peptidase gene (*lep B*) [38] were identified, respectively. However, no genes were predicted to be highly similar to the commonly presented immune genes found in the known circular bacteriocin gene clusters [37].

### 2.4. Evidences of Flexusin A Being a Novel Circular Bacteriocin Encoded by bfcir-1

As mentioned above, the antiSMASH and BAGEL4 databases mining allowed the discovery of the new circular bacteriocin gene *bfcir-1* with its encoding bacteriocin precursor peptide designed as BFCir-1 (mw = 6809.12 Da), correspondingly. One question thus arose: if flexusin A, the putative bacteriocin purified from the fermentate of R29-2, would be the mature bacteriocin encoded by *bfcir-1* and generated through posttranslational modification afterward (e.g., circularization as found in other circular bacteriocins) [39].

**Figure 6:**
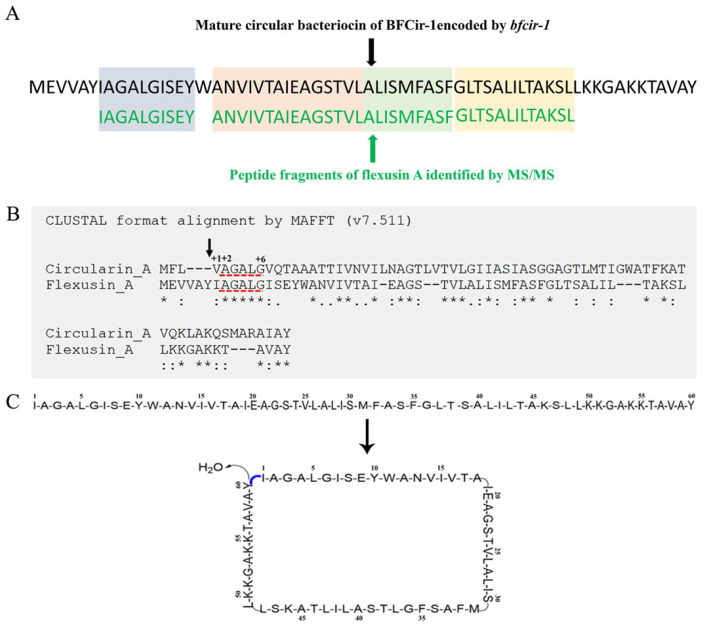
Analysis of the amino acid sequence of flexusin A. (**A**) Comparison of amino acid sequence encoded by *bfcir-1* gene with the MALDI-TOF MS/MS derived peptide fragment sequences of flexusin A. (**B**) Alignment of amino acid sequence of the precursor peptides of circularin A and flexusin A. Gap (−), identical amino acids (*), conservative substitutions (:), and weak conservative substitutions. The black arrow indicated the excision site of signal peptide of circularin A, demonstrating that Val at +1 position is the first amino acid at the N-terminal of the mature circularin A. The N-terminal amino acid sequence marked by red underline referred to the identical sequence of both circularin A and flexusin A. (**C**) Predicted mature circular structure of flexusin A after the loss of one water. The N-terminal amino acid (I_1_) and C-terminal amino acid (Y_60_) of linear flexusin A are dehydrated and condensed to form circular flexusin A by forming peptide bonds.

Shot-gun analysis was then applied to obtain sequence profile of flexusin A. The single band peptide recovered from Tricine-SDS-PAGE was cleaved by chymotrypsin, and derived peptide fragments were subjected to HPLC purification, followed by MS/MS, respectively. As shown in Figure S1, up to four sets of peptide sequences were successfully obtained by shot-gun analysis: IAGALGISEY, ANVIVTAIEAGSTVL, ALISMFASF, and GLTSALILTAKSL. Then, the shot-gun derived sequences were aligned with the predicted whole sequences of BFCir-1, the precursor peptide of the predicted circular bacteriocin. It was found that the four peptide fragments fitted in perfectly and were identical to the corresponding regions along the whole length of BFCir-1 (Figure 6A). Thus, it was clearly indicated that flexusin A was the mature circular bacteriocin encoded by *bfcir-1*, whereas BFCir-1 was the very precursor peptide of the bacteriocin. The precursor peptide sequence of flexusin A is MEVVAYIAGALGISEYWANVIVTAIEAGSTVLALISMFASFGLTSALILTAKSLLKKGAKKTAVAY.

A PubMed-based data search found that this is the first report regarding the production of bacteriocin by the *B. flexus* species.

Recently, our group characterized a novel circular bacteriocin velezin [34]. Almost around the same time, raffinocyclicin was reported as another new circular bacteriocin this year [40]. With velezin and raffinocyclicin included, thus far up to 25 circular bacteriocins have been characterized. The finding of flexusin A in this work has added 26th member to the circular bacteriocins family.

The peptide sequence of flexusin A was uploaded to MAFFT for sequence aligning with that of all the known circular bacteriocins (shown in Figure S2); derived data were applied for constructing the phylogenetic tree by using MEGA 7 (Figure S3). It was demonstrated that among circular bacteriocins known, circularin A was the most similar to flexusin A. However, the similarity between circularin A and flexusin A was even less than 50% (Figure 6B).

### 2.5. Determination of the Signal Peptide of Flexusin A

Usually, the bacteriocin precursor peptides contain a leader peptide (also known as signal peptide) located at the N-terminal to facilitate the secretion of bacteriocin extracellularly [41,42]. For some bacteriocins, e.g., lantibiotics and circular bacteriocins, there is an extra post-translational modification process at the end stage of biosynthesis, which concurred with the leader peptide cleaving from the precursor peptide [34,43,44,45,46,47,48,49,50]. For circular bacteriocins the featured post-translational modifications are characterized by the head-to-tail ligation of the backbone of these peptides [39].

Thus far, the biosynthetic mechanism of circular bacteriocins has not been fully elucidated, and the process encompassing leader peptide cleavage, core peptide circularization, and bacteriocin secretion remains poorly understood [39]. For the signal peptides of reported circular bacteriocin, the sequence conservativity is relatively low in amino acid composition and length, and so far no identical or conservative amino acids combination or sequence has been identified [48].

In flexusin A gene cluster, *lep B*, a signaling peptidase gene was identified (Figure 5), and it may be involved in the excision of flexusin A precursor peptide sequence. However, the signal peptide removal site was unable to be identified when the precursor peptide sequence of flexusin A was uploaded to SignalP database for leader peptide prediction.

As illustrated in Figure 6B, mature circularin A has six amino acid sequence VAGALG at the N-terminal of its mature peptide (from +1 to +6) with the short leader peptide MFL cleaved. Actually it was reported that the two short-side chain hydrophobic residues at positions +1 and +2 (Val and Ala in Figure 6B) of circularin A are required for the N-terminus to form active peptide derivatives, as their hydrophobic features are of crucial importance in interacting with (or binding to) the biosynthetic enzyme(s) [39]. And when the +1 amino acid Val was replaced by hydrophobic amino acid Ile, the mutated peptide still kept the antibacterial activity [39].

It was of interest that an identical five amino acid sequence AGALG (as underlined in red, Figure 6B) was found in the precursor peptides N-terminals for both circularin A and flexusin A. Actually, AGALG is exactly the fragment from +2 to +6 to the N-terminal of circularin A. As aforementioned, results derived by MAFFT analysis showed that circularin A was the circular bacteriocin most similar to flexusin A. Interestingly, in the case of circularin A, the amino acid situated in front of AGALG is Ile, which is also hydrophobic. Therefore, it was presumed that the fragment from +2 to +6 of the mature flexusin A should be AGALG, given the assumption that the N-terminal six amino acids MEVVAY at the precursor peptide as its leader peptide. Then, the hypothetic linear form peptide of flexusin A should be IAGALGISEYWANVIVTAIEAGSTVLALISMFASFGLTSALILTAKSLLKKGAKKTAVAY, with a calculated molecular mass of 6116.29 Da (60 amino acids in total, net charge of +3).

**Figure 7:**
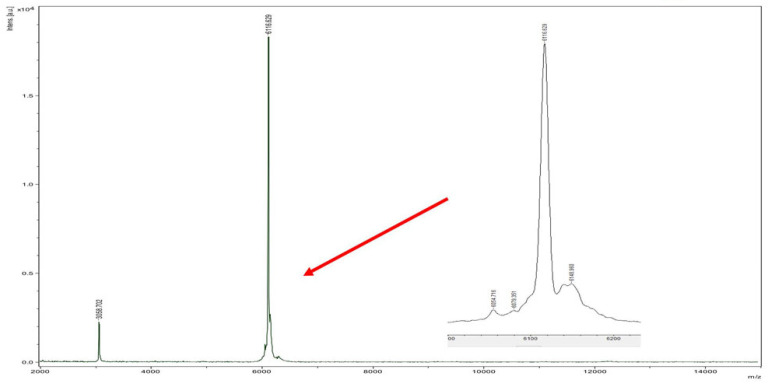
MALDI-TOF MS analysis of the peak at 35.527 min in the HPLC profile of Figure 2. The results confirmed that peptide in the peak was the linear form peptide of flexusin A.

During the HPLC purification of flexusin A, we detected a peptide peak adjacent to the peak of flexusin A, and it did not exhibit antibacterial activity (Figure 2). By applying MALDI-TOF MS, the molecular mass was determined to be 6116.629 Da (Figure 7), which almost 100% matched the value of 6116.29 Da, the calculated molecular mass of the assumed linear flexusin A after cleavage of the assumed leader peptide MEVVAY. These findings clearly support the inference that N-terminal MEVVAY in the flexusin A precursor peptide is its signal peptide.

Interestingly, the linear form of flexusin A exhibits near-complete loss of antibacterial activity. This phenomenon aligns with observations reported for two circular bacteriocins, gassericin A and enterocin AS-48 [51,52]. Cyclization has been identified as a critical determinant for preserving antimicrobial efficacy in these peptides. Notably, linear variants of both bacteriocins demonstrated significantly reduced bioactivities, showing 200- and 300-fold decreases compared to their circular counterparts, respectively.

Furthermore, the chemical capping modification, e.g., amidation of the C-terminal may influence the antimicrobial properties of leaner cationic antimicrobial peptides. C-terminal amidation has been shown to modulate biological activity and membrane interaction in cationic antimicrobial peptides, as exemplified by maculatin 1.1 [53]. Structural analyses revealed that C-terminal amidation enhances α-helix stability during micelle interactions, conferring both increased antibacterial potency and cytotoxicity compared to the acidic form of maculatin 1.1.

Future investigations should prioritize comprehensive structure-activity relationship studies of flexusin A. This would require: (1) Purified samples of both mature (circular) and linear forms. (2) Application of advanced structural characterization techniques (e.g., circular dichroism spectroscopy, high-resolution NMR). And, (3) Systematic evaluation of physicochemical parameters (charge distribution, hydrophobicity) and their correlation with antimicrobial function.

With the loss of one water (18 Da) in the cyclization step during post-translational modification, the calculated MW of mature flexusin A should be 6098.29 Da (Figure 6C). Surprisingly again, this calculated MW perfectly matches the value of 6098.4308 Da, the LC-MS measured molecular mass of purified flexusin A. Taken together, it was concluded that the first six amino acids sequence MEVVAY is the leader peptide of flexusin A.

In the biosynthesis of circular bacteriocin, the cleavage and circularization may be two separate processes, which can be supported by the detection of the un-circularized linear peptide form, as exemplified by the case reported in garvicin ML [54]. In this work, the linear form peptide of flexusin A was also detected from the peptide extract of the fermentation broth of R29-2. This result simply infers that during flexusin A biosynthesis the leader peptide cleavage and circularization of the linear peptide may also be two separate processes, and both the linear and circular form peptides of flexusin A could be secreted separately to the extracellular environments.

### 2.6. Three-Dimensional Model Prediction of Flexusin A

In general, circular bacteriocins usually contain 58–70 amino acids and have 4–5 α-helices along the peptide chain [39]. The secondary structure of circular bacteriocin flexusin A were predicted by Jpred3 and TMHMM-2.0 databases. Since the N-terminal and C-terminal of flexusin A are closed into loops, different amino acid sites were selected as the N-terminal of the linear peptide when the linear sequence was uploaded to the database for secondary structure prediction. It was predicted that after excision of the leader peptide the secondary structure of flexusin A contains five α-helical structures and no β-folded structures, which is consistent with the secondary structural features of the circular bacteriocins reported.

**Figure 8:**
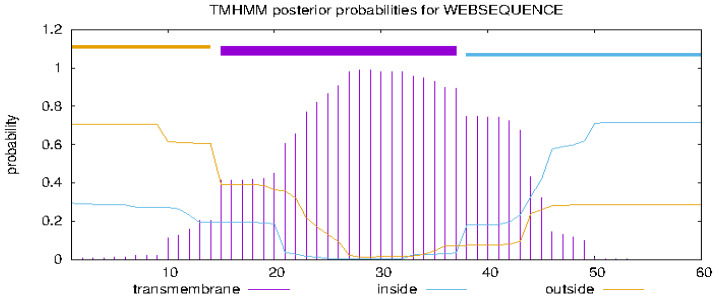
Transmembrane region prediction of circular bacteriocin flexusin A by secondary structure analysis using TMHMM-2.0.

The circular bacteriocins are amphiphilic and generally positively charged overall, and this feature allow them to be able to bind the negatively charged target cell membrane of the Gram-positive [39]. The hydrophobic transmembrane regions of the known circular bacteriocins are also closely related to the mechanism of actions, e.g., the insertion and the followed membrane destabilization in the cell membrane layers through hydrophobic interactions [48]. The transmembrane region of mature flexusin A was predicted by applying TMHMM-2.0 database. It was found that amino acids at positions 1-25 and 29-51 comprised two segments of transmembrane regions with up to 80% of amino acid sequences situated in the transmembrane region (Figure 8). This rate is higher than that of velezin, the circular bacteriocin we characterized recently, with which the proportion of amino acids in the transmembrane region was 27% [34].

**Figure 9:**
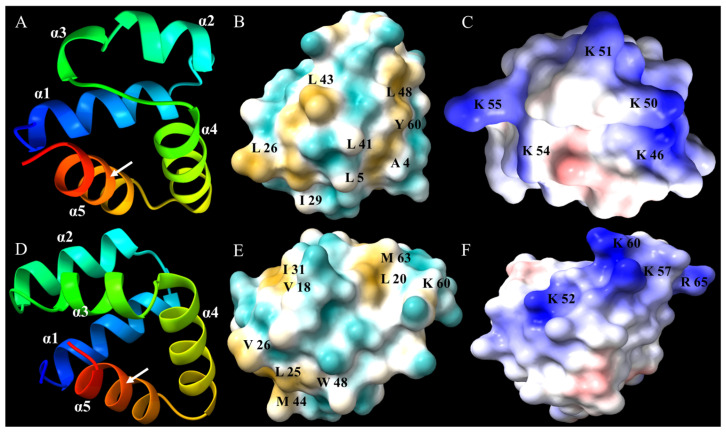
Three-Dimensional model prediction of flexusin A and circularin A by AlphaFold2. (**A**,**D**) Ribbon diagram of flexusin A and circularin A with five helices (α1, α2, α3, α4 and α5), respectively, and the positions of N-to-C connection were indicated by the white arrows in the figure. (**B**,**E**) Hydrophobic amino acids distribution on the surface of flexusin A and circularin A, with the hydrophobic regions marked in yellow and the surface positioned amino acids labeled in black, respectively. (**C**,**F**) Electrostatic surface potential features of flexusin A and circularin A, indicating positively charged amino acid (labeled in black) located in semi-continuous positive charged regions (marked in blue), respectively. The small negative charge regions were marked in red.

The sequence of flexusin A was uploaded to the AlphaFold2 database for structure prediction modeling. Its close homolog circularin A was also uploaded to the AlphaFold2 database as a reference. The modeling data were further analyzed by applying Chimera X 1.9 software for 3D model diagraming (Figure 9). The results of model diagrams showed that both flexusin A and circularin A contain 5 α-helices (Figure 9A,D).

Some circular bacteriocins have a recuring three-dimensional structural conformation known as a saposin-like fold, which is structurally a motif of multiple helices, and the circularization site is typically found within an α-helical structure [19,55]. It has been well recognized that the saposin fold motif-like structural conformation is crucial to the antibacterial activities of a panel of circular bacteriocins, as well as to the superior stabilities against thermal stress, pH variation, and the resistance to degradation by proteolytic enzymes [47,48,56]. As shown in Figure 9A, the saposin fold conformation was also observed in the 3D model of flexusin A. It was arranged by five amphipathic α-helices in a distinctly compact architecture. The simulation results of hydrophobic surfaces show that, in general, hydrophilic and hydrophobic amino acids were relatively evenly distributed on the bacteriocin surface (Figure 9B). However, like in the case of velezin [34], some hydrophobic patches were observed at the surface of the spherical structure (marked in yellow in Figure 9B), which may be involved in the hydrophobic interactions and amphiphilic features of the bacteriocins, as reported elsewhere [47,48].

In predicating the electrostatic surface potential of flexusin A by AlphaFold2, it was noticed that up to five lysine residues were positioned at the surface and partially clustered, allowing the formation of a semi-continuous positive charged region (marked in blue, Figure 9C). Just as reported elsewhere [48], the positive charged region may be essential for flexusin A to exert its antibacterial activity.

It is of interest to note that although the sequence similarity between circularin A and flexusin A was even less than 50%, the two bacteriocins did share some similar spatial structural conformation, such as the saponin-like fold consisting of five α-helices with the circularization site located in one α-helical structure (Figure 9A,D), the evenly distributed hydrophilic and hydrophobic amino acids on the surfaces of both bacteriocins (Figure 9B,E) and the charge distribution patterns (Figure 9C,F).

### 2.7. MIC of Flexusin A

The purified flexusin A was examined for its potency in inhibiting the Gram-positive pathogens in “ESKAPE”, including *Staphylococcus aureus*, *Enterococcus faecium* and *Enterococcus faecalis.* The protein concentration of the stock solution of flexusin A (256 μg/mL) was determined to be 250.37 μg/mL based on the BCA method, indicating its purity, attaining 98%. It was found that purified flexusin A had excellent activities against the three pathogens listed above, with minimum inhibitory concentrations (MICs) as low as at µg/mL level (Table 2). For the case of *E. faecium* CICC 10004, it was noticed that MIC of flexusin A was even lower than that of ampicillin. These results indicated that flexusin A may present comparable activity to ampicillin in inhibiting the Gram-positive pathogens included in “ESKAPE” under the same conditions. In addition, flexusin A also demonstrated decent inhibition effect against the famous foodborne pathogen *L. monocytogenes* ATCC 19115.

### 2.8. Mechanism of Action

**Figure 10:**
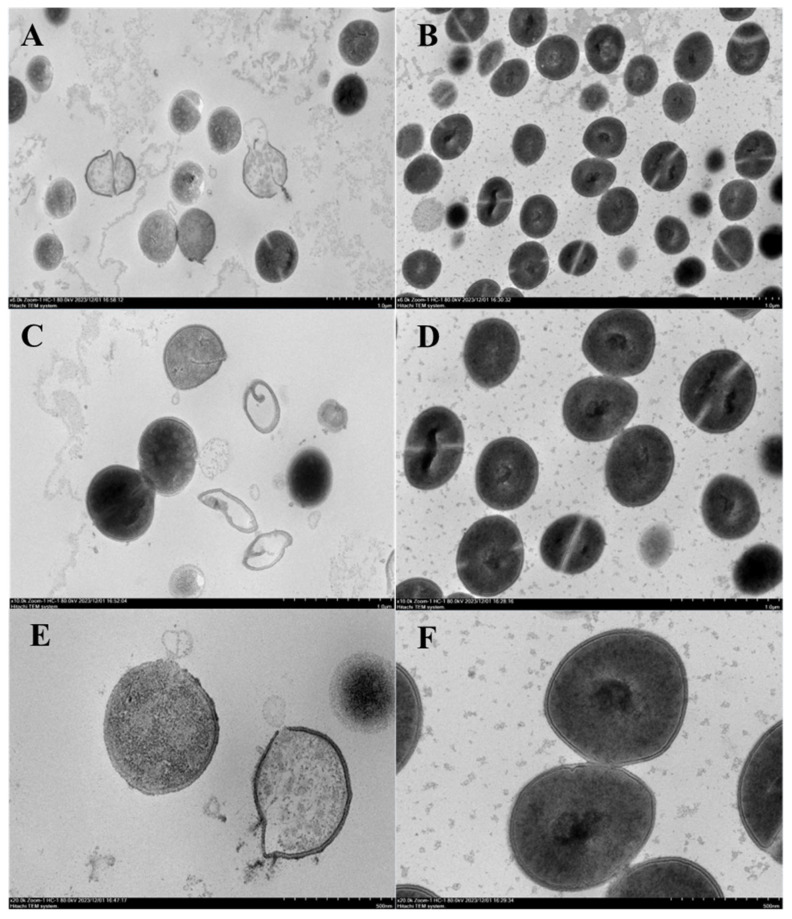
TEM ultrathin section observation of *S. aureus* after flexusin A treatment. Pictures on the left panel demonstrated the cells images of *S. aureus* treated by flexusin A at 1× MIC, with magnification of 6 k (**A**), 10 k (**C**), and 20 k (**E**), respectively. Pictures on the right panel demonstrated the cells images of *S. aureus* in the control, with magnification of 6k (**B**), 10 k (**D**), and 20 k (**F**), respectively.

To determine the mechanism of action, 1× MIC flexusin A-treated cells of *S. aureus* ATCC 6538 was checked by transmission electron microscopy (TEM) ultra-thin section observation. As observed, in the control group the cell boundaries were complete, smooth, and clear, with the density of cytoplasmic materials uniformly distributed (Figure 10B,D,F). For the treatment receiving flexusin A, membrane destabilization, shrinkage, cleavage in the marginal structure, and even the collapse of *S. aureus* ATCC 6538 cells was clearly observed (Figure 10A, magnification of 6 k). The more defined images (Figure 10C, magnification of 10 k) demonstrated that the interior of some cell was completely transparent, with whole cells crumbled and disintegrated. And the much-magnified pictures provided the details of the membrane damage as well as the events depicting cell contents leakage through the damaged membrane (Figure 10E, magnification of 20 k). Taken together, the direct TEM observation results demonstrated that the antibacterial mechanism of flexusin A was mainly through destabilizing the infrastructure of cell membrane through forming permanent damage to the membrane that caused the leakage of cell contents and the eventual collapse of the cells.

### 2.9. Stability of Flexusin A

**Figure 11:**
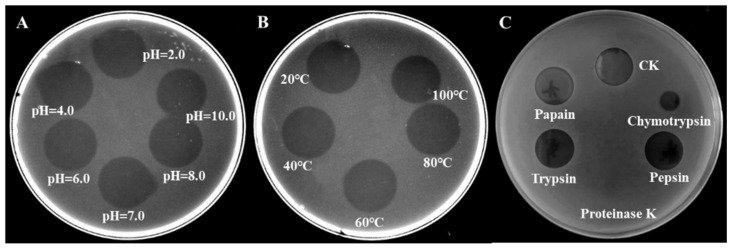
The stability tests showing the remaining activities of flexusin A when it was subjected to different conditions of pH (**A**), temperature (**B**) as well as to the challenges by different proteases (**C**).

Circular bacteriocins are well known for their high pH and thermal stability, as well as for the resistance to certain proteolytic enzymes [47]. The effects of temperature, pH and proteolytic enzymes on the stability of flexusin A were shown in Figure 11. The results demonstrated that flexusin A was stable over a pH range from 2.0 to 10.0. And it exhibited decent thermal stability when treated for 30 min from 20 to 100 °C. Flexusin A also demonstrated certain resistances to proteolytic enzymes. It was tolerant to trypsin, pepsin and papain, but was sensitive to protease K and chymotrypsin.

## 3. Materials and Methods

### 3.1. Samples and Microorganism Cultivation

The seamount samples including seawater and mud, were collected from the Caroline Seamount ecosystem in the Western Pacific Ocean (139°51′ E–140°24′ E, 10°3′ N–10°57′ N). In the experiment, LBH medium (yeast powder 5 g, sodium chloride 30 g, tryptone 10 g, seawater 30 g, distilled water 1000 mL, pH6.5, sterilization) was used to isolate bacteria from seamount samples.

All indicator microorganisms used in this study were listed in Table 1. All chemicals and reagents were of analytical grade and purchased from the sources commercially available.

### 3.2. Screening for Marine Bacterial Isolates with Antibacterial Activity

The pure cultures of the bacteria isolated from seamount samples were spot onto LBH plates and cultured at 28 °C for 1 day, allowing colonies to develop. *Staphylococcus aureus* ATCC 6538 was used as an indicator strain for antimicrobial assays. To identify antimicrobial activity of the testing colonies, the overlay assays were performed by overlaying plates using soft agar seeded with broth of *S. aureus* ATCC 6538 [57]. The antibacterial zone was observed, and its diameter was recorded (Table 1). Bacteria with clear antibacterial zones were subjected to further antibacterial spectrum analysis.

### 3.3. 50 L Scale Pilot-Scale Submerged Fermentation of B. flexus R29-2

*B. flexus* R29-2 seed was prepared in a flask using LBH medium cultured at 32 °C for 48 h, and then it was used to inoculate the 50 L fermenter (FZ-EI 50L automatic fermenter (made by Jiangsu Fengze biological engineering equipment Manufacturing Co., Ltd., Zhenjiang, China) containing a 30 L medium with inoculum size of 1%. Initial fermentation conditions of temperature, pH, and agitation aeration were set at 32 °C, 6.5, 150 rpm, respectively. An aseptic antifoaming agent was added during the fermentation process for foaming control. During fermentation, the pH was controlled to be no less than 6.5 by automatic addition of ammonia water. The ventilation volume and speed were adjusted automatically in response to the change in dissolved oxygen during the fermentation process to meet the demand of the dissolved oxygen setting. A glucose solution was sterilized separately and was added into the fermentation broth through a programmed feeding profile. During fermentation, the bacteriostatic activity of the fermentation broth was determined by the overlay method, as aforementioned.

### 3.4. Isolation and Purification of the Antibacterial Substance

The antibacterial substance was isolated by using macroporous resin absorption, as reported [12], followed by extraction with trichloromethane. The fermentation broth was centrifuged at 10,000× *g* for 20 min at 25 °C to spin down the cells. The pooled supernatant was dynamically passed in a column filled with macroporous resin (D4006, Tianjin Nankai University resin Co., Ltd., Tianjin, China) at a flow rate of 10 mL/min, allowing the complete absorption of the bacteriocin to the resin. The column was then eluted 3 times with a bed size of distilled water, 30% ethanol and 75% ethanol, respectively. Each eluent fraction was successively collected for antibacterial testing. Fraction with activities was then concentrated by rotary evaporation at 40 °C under vacuum. The concentrate was further extracted against trichloromethane of equal volume by fully mixing and centrifugation, leaving impurities in the water phase and trichloromethane phase while allowing the bacteriocins alike enriched in the intermediate phase. A crude antibacterial solution was obtained by dissolving the spin-down of the intermediate phase using ethanol, and it was further concentrated by vacuum evaporation.

Crude antibacterial concentrate was further purified by HPLC with a Phenyl Ether column (4.6 × 250 mm, 5 μm, Yuexu Technology (Shanghai) Co., Ltd., Shanghai, China). The HPLC system used was CHROMASTER ORGANIZER of Hitachi (Hitachi High-tech Science Corporation, Tokyo, Japan) consisting of a Chromaster 5110 pump, 5210 auto sampler, Chromaster 5310 column oven, and a Chromaster 5420 UV-Vis monitor. The mobile phase consisted of (A) acetonitrile (containing 0.1% trifluoroacetic acid, TFA) and (B) HPLC-grade water containing 0.1% TFA. The elution profile was set as follows: 0–10 min, A: 40–55%; 10–15 min, A: 55–70%; 15–25min, A: 70–85%; 25–30 min, A: 85–90%; 30–40 min, A: 90–100%; 40–45 min, A: 100%. For separation, the sample injection volume was 20 μL and the elution rate was set at 1 mL/min, respectively. Elution was monitored at a wavelength of 220 nm, and fractions were collected manually for antimicrobial activity bioassay. Fractions with antimicrobial activity were collected, pooled, concentrated and further repurified using the same conditions as described. Pooled antibacterial fractions were lyophilized and reconstituted. The antimicrobial agent derived was referred to as flexusin A.

Each peak elution was collected separately for antibacterial activity measurement using the overlay method as mentioned. Purified antibacterial sample stock was prepared by pooling the fractions with activity and then concentrated by evaporating residual solvent.

### 3.5. Tricine-SDS-PAGE of Purified Flexusin A Sample

Samples containing flexusin A were subjected to Tricine-SDS-PAGE check. Tricine-SDS-PAGE analysis was performed by the methods as used elsewhere [57,58] with minor modifications. In the experiments, 4% and 10% of acrylamide were used for the stacking and separation of gels, respectively, with gels running at 30 V for 30 min and then at 150 V for 1 h. The gels were visualized by coomassie blue G-250 staining.

### 3.6. Mass Spectrometry

LC-MS of purified sample of flexusin A was conducted using 1290 Infinity II UHPLC/6460 QqQ MS (Agilent, Santa Clara, CA, USA) with a column Phenyl-Ether (4.6 × 250 mm, 5 μm). The ionization mode was electrospray and positive ion mode [59]. The mass detector used was a four-stage mass analyzer, and the mass spectrum scanning range was 0–3000 *m*/*z*. HPLC conditions: Solvent A, acetonitrile (0.5% formic acid) and solvent B, water (0.5% formic acid); flow rate of 0.3 mL/min monitoring wavelength at 220 nm.

For MALDI-TOF analysis, an autoflex MALDI-TOF mass spectrometer (Bruker, Karlsruhe, Germany) was used by following the protocols [60].

### 3.7. Whole Genome Analysis of B. flexus R29-2

Genomic DNA of R29-2 was extracted according to the operating instructions of the DNA extraction kit (Tianjin Gamei Huzhong Technology Co., Ltd., Tianjin, China), and it was sent to Shanghai Maiji Biotechnology Co., Ltd. (Shanghai, China) for genome sequencing by Illumina and PacBio platforms, followed by De novo genome assembly to obtain the assembled genome of the strain [40]. The genes relevant to the metabolites, including the genes clusters of bacteriocin biosynthesis, were predicted and annotated by uploading the genebank files of R29-2 to BAGEL4 (http://bagel4.molgenrug.nl/index.php, accessed on 20 May 2024) and AntiSMASH (https://antismash.secondarymetabolites.org/, accessed on 20 May 2024) databases, respectively [35,36]. The putative functions of genes associated with bacteriocin biosynthesis were furtherly predicted by using databases of NR, Swiss-Prot, Pfam, EggNOG, GO, and KEGG. And the protein information encoded by the genes, such as molecular mass, isoelectric points, aliphatic index (AI), and grand average of hydropathicity were determined using ProtParam (https://web.expasy.org/protparam/, accessed on 20 May 2024).

### 3.8. Shot-Gun Proteomics Analysis and Structure Prediction of Flexusin A

The amino acid composition and partial sequence of flexusin A were identified by shot-gun proteomics analysis and genome sequencing of producing bacteria R29-2 [61,62]. Expansively, the Tricine-SDS-PAGE derived single band of flexusin A was recovered by decolorization, reduction, and dehydration, then followed by digestion using chymotrypsin (0.1 μg/μL) and the gel was covered with 25 mmol/L NH_4_HCO_3_ solution containing 10% acetonitrile at 37 °C for overnight, and lastly the aqueous solution (5% TFA, 67% acetonitrile) was used to extract the peptide solution [63]. Derived enzymatic hydrolysate was sent to Sangon Biotech (Shanghai, China) for MALDI-TOF/TOF secondary mass spectrometry analysis. The sequences of different fragments analyzed by mass spectrometry were aligned with the genome sequencing results of the producing bacterium R29-2 to determine the primary structure of flexusin A. The peptide sequence of flexusin A was uploaded to MAFFT for sequence aligning with that of all the known circular bacteriocins, and derived data were applied for constructing the phylogenetic tree by using MEGA 7. Prediction of the 3D model features of flexusin A was conducted by using SignalP—5.0 database (https://services.healthtech.dtu.dk/service.php?SignalP-5.0, accessed on 20 May 2024). The possible transmembrane regions in flexusin A were predicted by TMHMM-2.0 (https://services.healthtech.dtu.dk/service.php?TMHMM-2.0, accessed on 20 May 2024). The secondary structures of α-helix, β-fold and random curling in mature circular bacteriocin flexusin A were predicted by using the Jpred database (http://www.compbio.dundee.ac.uk/jpred/, accessed on 20 May 2024). The secondary and tertiary structures of flexusin A and its close homolog circularin A were calculated and predicted through AlphaFold2 (https://alphafold.com/, accessed on 20 May 2024) [64], and the 3D model maps were generated using ChimeraX 1.9 software [34]. The charged and hydrophobic surfaces region associated with bacteriocin antibacterial action were distinguished by different colors.

### 3.9. MIC Values of Flexusin A

An array of indicator strains (*S. aureus* ATCC 6538, *E. faecalis* CICC 21605, *E. faecium* CICC 10004 and *L. monocytogenes* ATCC 19115) were used to determine the MIC of flexusin A by the methods, as reported [65]. Briefly, the purified flexusin A was 2-fold serially diluted with sterile water, then 10 μL diluted solution was added to 90 μL fresh medium, which containing approximately 1 × 10^6^ CFU/mL indicator strains in 96-well plates, and the mixture was incubated at 37 °C for 24 h. The purified powder of flexusin A was reconstituted at 256 μg/mL to serve as the stock solution. It was then used for the antibacterial activity assay over a range of 256, 128, 64, 32, 16, 8, 4, 2, 1, 0.5, 0.25, and 0.125 μg/mL. The protein concentration of the flexusin A stock solution was determined by the BCA method (BCA Protein Assay Kit, Sangon Biotech)”. Water was used as a negative control, whereas ampicillin (Solarbio, Beijing, China) was used as positive control. All assays were performed in triplicate. MIC was defined as the lowest concentration of flexusin A that inhibited indicator growth, as no visible microbial growth was observed.

### 3.10. Observations of Ultrathin Section Under Transmission Electron Microscopy (TEM)

The cell integrity of *S. aureus* after flexusin A treatment (at 1× MIC) was observed by TEM ultrathin section [62]. Flexusin A sample was added to the bacterial solution with a cell concentration of about 10^6^ CFU/mL making the final concentration at 1× MIC, whereas the bacterial solution with equal volume of sterile water was used as negative control. After incubation at 37 °C for 1 h, the bacterial cells were spun down at 3000 r/min for 10 min and rinsed twice with 0.1M phosphate buffer (pH = 7.2). The bacteria cells were finally spun down to the bottom of a 1.5 mL pointy bottom centrifuge tube, and then a fresh fixing solution (2.5% glutaraldehyde) was slowly added along the EP tube to fix the cell at 4 °C overnight. The fixed sample was sent to the Institute of Biophysics, Chinese Academy of Sciences for sectioning and observation.

### 3.11. Sensitivity to Heat, pH, and Degradative Enzymes

The HPLC purified bacteriocin samples were made readily soluble in neutral water, and the stock solution was then tested for sensitivity to heat, pH changes, and degradative enzymes [12]. The qualitative spot-on-lawn bioassay was used to monitor changes in antimicrobial potency after treatments mentioned. For thermal stability testing, aliquots of the HPLC-purified peptide samples were exposed to 30 °C, 40 °C, 50 °C, 60 °C, 70 °C, 80 °C, 90 °C, and 100 °C for 30 min, respectively. For the pH stability test, aliquots of the stock solutions were adjusted to pH 2.0, 3.0, 4.0, 5.0, 6.0, 7.0, 8.0, and 9.0, followed by incubation at 25 °C for 2 h.

Sensitivity to various degradative enzymes was determined using trypsin, papain, pepsin, chymotrypsin, and protease K at a final concentration of 1 mg/mL, respectively. All enzymes were purchased from Sigma, and their solutions were prepared in 25 mM phosphate buffer, pH 7.0 (pepsin, pH = 2.0). Solutions of the antimicrobial peptide were prepared in the same sterile buffer. After mixing the enzymatic reaction solutions were incubated at 37 °C for 4 h before residual antimicrobial activity measurement.

## 4. Conclusions

In this work, for the first time we report the discovery of a novel circular bacteriocin flexusin A from the fermentate of marine bacterium *B. flexus* R29-2. A set of pilot scale submerged fermentation techniques was established for the bacteriocin production, and then the peptide was successfully isolated and purified out of the fermentation broth. Sequence alignment facilitated with shot-gun measurement versus the gene database predication enabled the full characterization of flexusin A regarding the characterization of the precursor peptide, leader peptide as well as the mature circular bacteriocin. It is worth mentioning that thus far up to 25 cases of circular bacteriocins have been fully characterized. The characterization of flexusin A in this work allowed the addition of the 26th member with marine origin to the circular bacteriocins family.

Flexusin A exhibited a narrow yet strong antibacterial spectrum against Gram-positive pathogens included in the ones of ESKAPE as well as the foodborne pathogen *L. monocytogenes*. TEM observations have confirmed that flexusin A causes the destruction of *S. aureus* ATCC 6538 cells through membrane destabilization and the ensued release of cell contents of the target. Flexusin A is water soluble, and it exhibits excellent pH and thermal stabilities as well as resistance to the challenge of proteolytic enzymes. In addition, as exemplified in this work, flexusin A is suitable to be produced by submerged fermentation at pilot scale even by the original producer strain *B. flexus* R29-2, and the downstream isolation and purification can be easily achieved and established via cost-effective manipulations. Taken together, these findings reveal that flexusin A may serve as a promising candidate in the arsenal used in the campaign against emerging drug-resistant Gram-positive pathogens.

## Figures and Tables

**Table 1:** Antimicrobial spectrum of *B. flexus* R29-2.

Strains Tested	Source	Medium and Temperature (°C)	Inhibition Zone
Gram-positive bacteria			
*Staphylococcus aureus*	ATCC 6538	LB, 37 °C	+++
*Enterococcus faecalis*	CICC 21605	MRS, 37 °C	++
*Enterococcus faecium*	CICC 10004	MRS, 37 °C	++
*Listeria monocytogenes*	ATCC 19115	BHI, 37 °C	+++
*Exiguobacterium indicum*	from lab stock	LB, 37 °C	++
*Bacillus aryabhattai*	from lab stock	LB, 37 °C	++
*Bacillus amyloliquefaciens*	from lab stock	LB, 37 °C	++
*Bacillus pumilus*	from lab stock	LB, 37 °C	+
*Bacillus cereus*	from lab stock	LB, 37 °C	++
*Clostridium perfringens*	from lab stock	RCM, 37 °C	−
*Bacillus cereus*	from lab stock	LB, 37 °C	+
Gram-negative bacteria			
*Aeromonas hydrophila*	from lab stock	LB, 37 °C	−
*Klebsiella pneumoniae*	from lab stock	LB, 37 °C	−
*Escherichia coli*	from lab stock	LB, 37 °C	−
*Vibrio parahaemolyticus*	ATCC17802	LB, 37 °C	−
*Pseudomonas aeruginosa*	from lab stock	LB, 37 °C	−
Fungi			
*Candida albicans*	from lab stock	YPD, 28 °C	−
*Aspergillus versicolor*	from lab stock	PDB, 28 °C	−

LB, Luria–Bertani; MRS, de Man, Rogosa and Sharpe; BHI, brain heart infusion; RCM, reinforced clostridium medium; YPD, yeast extract peptone dextrose medium; PDB, potato dextrose broth; +, indicating the diameter of inhibition zone ≤ 10 mm; ++, indicating the diameter of inhibition zone > 10 mm but ≤20 mm; +++, indicating the diameter of inhibition zone >20 mm; −, indicating no antibacterial activity observed.

**Table 2:** MIC tests * of Flexusin A.

Indicator Strains	FlexusinA MIC/(µg/mL)	Amp ** MIC/(µg/mL)
*S. aureus* ATCC 6538	1	0.4
*E. faecalis* CICC 21605	16	>51.2
*E. faecium* CICC 10004	32	1.6
*L. monocytogenes* ATCC 19115	2	0.0625

* MICs were determined by broth microdilution in 96-well in triplicate. ** Amp, ampicillin.

## Data Availability

The data presented in this study are available on request from the corresponding author.

## Supplementary Materials

The following supporting information can be downloaded at: https://www.mdpi.com/article/10.3390/md23030095/s1, Figure S1: MS/MS analysis of flexusin A peptides obtained by chymotrypsin digestion of flexusin A. Figure S2: Amino acid alignment of the mature circular bacteriocins known (MAFFT v7.4.89). Gap (−), the regions marked in black are highly homologous sequences, the gray marked region is slightly less conservative, and unshaded areas are regions of variability between circular bacteriocins. Figure S3: Maximum likelihood based on phylogenetic evolutionary tree of known 25 circular bacteriocins through MAFFT alignment with flexusin A included.
