# Supplementary material for: Characterization of Flexusin A, a Novel Circular Bacteriocin Produced by Marine Bacterium Bacillus flexus R29-2
Source: Mar Drugs. 2025 Feb 21;23(3):95. doi: 10.3390/md23030095 (PMC11943950; doi:10.3390/md23030095)

Figure S1. MS/MS analysis of flexusin A peptides obtained by chymotrypsin digestion of flexusin A. (A) IAGALGISEY, (B) ANVIVTAIEAGSTVL, (C) ALISMFASF and (D) GLTSALILTAKSL.

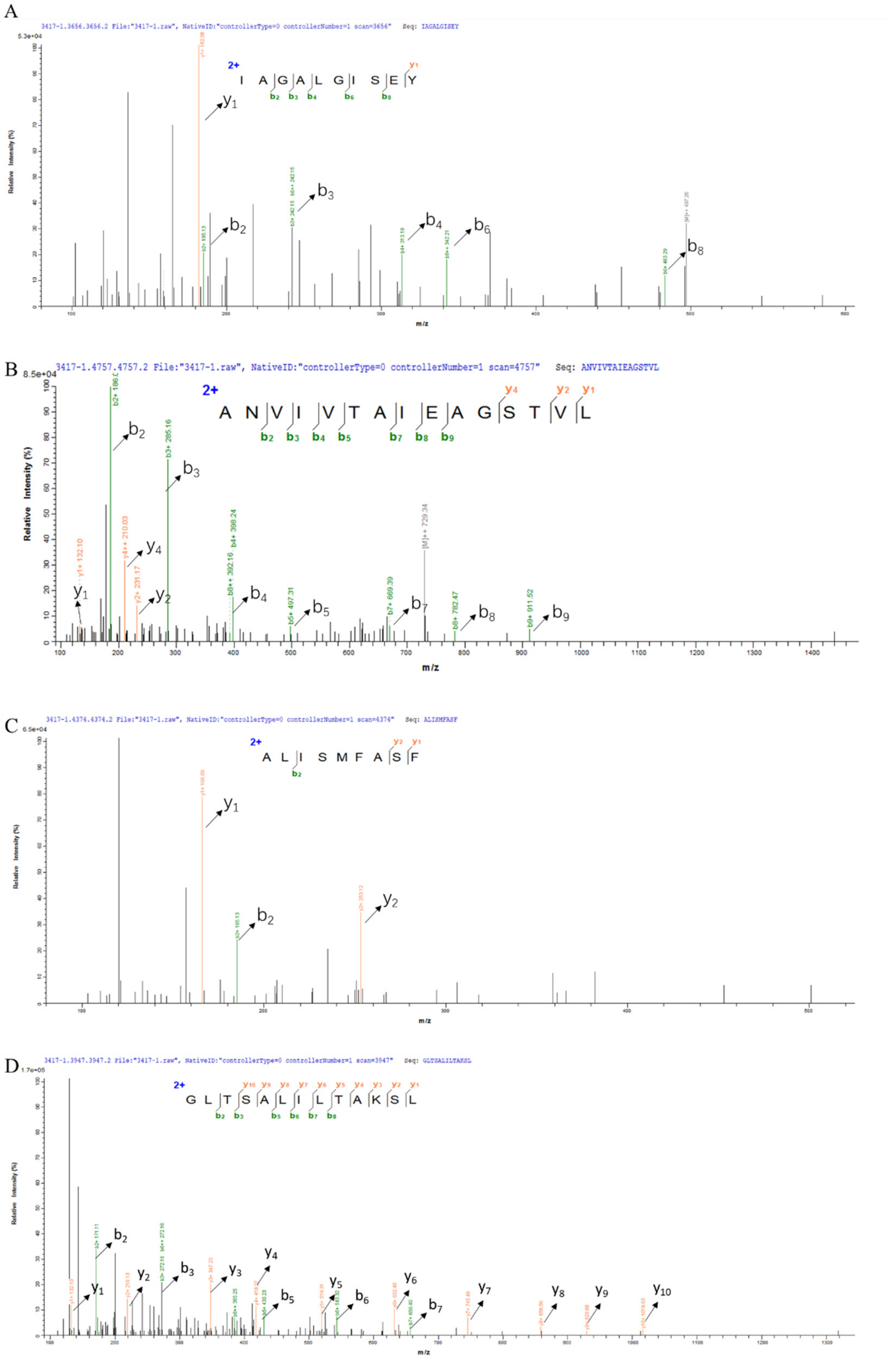

Figure S2. Amino acid alignment of the mature circular bacteriocins known (MAFFT v7.4.89). Gap (−), the regions marked in black are highly homologous sequences, the gray marked region is slightly less conservative, and unshaded areas are regions of variability between circular bacteriocins.

```
Flexusin_A        : ----------IAGALGI--SEY-WANVIVTAIEAGSTVLALISMFASFGLT-SALIL-----------TAKSLLKKGAKKT---AVAY : 60
Raffinocyclicin   : -----------LVATGM--GAG-TAASVVDAIFGGLTIASALSIYAGLAGG-AAWFLEN---------GLKTLIKWAGKRT---IISW : 61
Aureocyclicin_418 : -----------LTGLGI--GTG-MAATIINAISVGLSAATILSLISGVASG-GAWVLA----------GAKQALKEGGKKA---GIAF : 60
Bacicyclicin_xin- : -----------LTGIGI--GDG-TAATIVNWIMWGMSAATILSLISGVASG-GAWILA----------GAREALKAGGKKA---AIAW : 60
Garvicin_ML       : -----------LVATGM--AAG-VAKTIVNAVSAGMDIATALSIFSGAFTA-AGGIMA----------LIKKYAQKKLWKQ---LIAA : 60
Carnocyclin_A     : -----------LVAYGI--AQG-TAEKVVSLINAGLTVGSIISIIGGVTVG-LSGVFT----------AVKAAIAKQGIKK---AIQL : 60
Pallidocyclin     : ----------LLVAYGI--NET-TANTIAAFLDAGLGIASFLTLITGAGLT-LKALQIA--------------LRAGGKKA---VVA- : 56
Enterocin_AS48    : ----------MAKEFGI--PAA-VAGTVINVVEAGGWVTTIVSIITAVGSG-GLSLLAA-AGRESIKAYLKKEI-KKGKRA---VIAW : 69
Enterocin_AS48RJ1 : ----------MAKEFGI--PAA-VAGTVINVVVAGGWVTTIVSIITAVGSG-GLSLLAA-AGRESIKAYLKKEIKKKGKRA---VIAW : 70
Pumilarin         : ----------LAKEFGI--PGS-VAAVVINVVEAGGAVTTIVSIITAVGSG-GLSLIAA-AGKETIRQYLKNEIKKKGRKA---VIAW : 70
Cerecyclin        : VVS-------KLGWTGI--NIGTTANALIGALMTGSDIWTAISVAGLAFGG-GIGTAISTIGRK----AIMEMVEKVGKKK---AAQW : 71
Amylocyclicin     : ----------LASTLGI--STA-AAKKAIDIIDAASTIASIISLIGIVTGA-GAISYAI-------VATAKTMIKKYGKKY---AAAW : 64
Amylocyclicin_CMW : ----------LASTLGI--SAA-AAKKAIDIIDAASTIASIISLIGIVTGA-GAISYAI-------VATAKTMIKKYGKKY---AAAW : 64
Enterocin_NK-5-3B : ----------LTANLGI--SSY-AAKKVIDIINTGSAVATIIALVTAVVGG-GLITAGI-------VATAKSLIKKYGAKY---AAAW : 64
Altitudin_A       : ----------LATNLGI--SRK-TAYAAIGVIMTTGDVLTILSLIAVVLGGTGLVTAAM-------VATAKKLATKHGKKY---AAEW : 65
Uberolysin        : ----------LAGYTGI--ASG-TAKKVVDAIDKGAAAFVIISIISTVISA-GALGAVS-ASADFIILTVKNYISRNLKAQ---AVIW : 70
Circularin_A      : ----------VAGALGV--QTA-AATTIVNVILNAGTIVIVLGIIASIASG-GAGTLMT-IGWA----TFKATVQKLAKQSMARAIAY : 69
Lactocyclicin_Q   : ----------LIDHLGA--PRW-AVDTIIGAIAVGNLASWVLALVPGPGWA-VKAGLA----------TAAAIVKHQGKAA---AAAW : 61
Leucocyclicin_Q   : ----------LVNQLGI--SKS-LANTIIGAIAVGNLASWLLALVPGPGWA-TKAALA----------TAETIVKHEGKAA---AIAW : 61
Gassericin_A      : ----------IADQFGIHLATG-TARKLIDAMASGASIGIAFAAILGVTLP-AWALAAA---------------GALGATA---AIYW : 58
Acidocin_B        : ----------IADQFGIHLATG-TARKLIDAVASGASIGIAFAAILGVTLP-AWALAAA---------------GALGATA---AIYW : 58
Plantaricyclin_A  : ----------IAKQFGVHLTTS-LTQKAIDLISAGSSIGIVAAAVLGVTLP-AWAVAAA---------------GALGGTA---AIVW : 58
Plantacyclin_B12A : ----------IARQFGVHLTTK-LTQKAIDLISSGASIGIVAAVILGVTLP-GWAVAAA---------------GALGGTA---AIVW : 58
Butyrivibriocin_A : ----------IADKMGIQLAPA-WYQDIVNWVSAGGTITTGFAIIVGVTVP-AWIAEAA---------------AAFGIAS---AIYF : 58
Paracyclicin      : ----------IANKLGIHLAPG-WYQDMVNYVSAGGSIAGAFSVVAGVTLP-AWIVPIA---------------TAFGAVS---AIYF : 58
Velezin           : AAHAFSANAELASTLGI--STA-AAKKAIDIIDAASTIASIISLIGIVTGA-GAISYAI-------VATAKTMIKKYGKKY---AAAW : 74
```

Figure S3. Maximum likelihood based on phylogenetic evolutionary tree of known 25 circular bacteriocins through MAFFT alignment with flexusin A included. Bootstrapped *1,000 replicates. Flexusin A and circularin A were highlighted by a red dotted box.

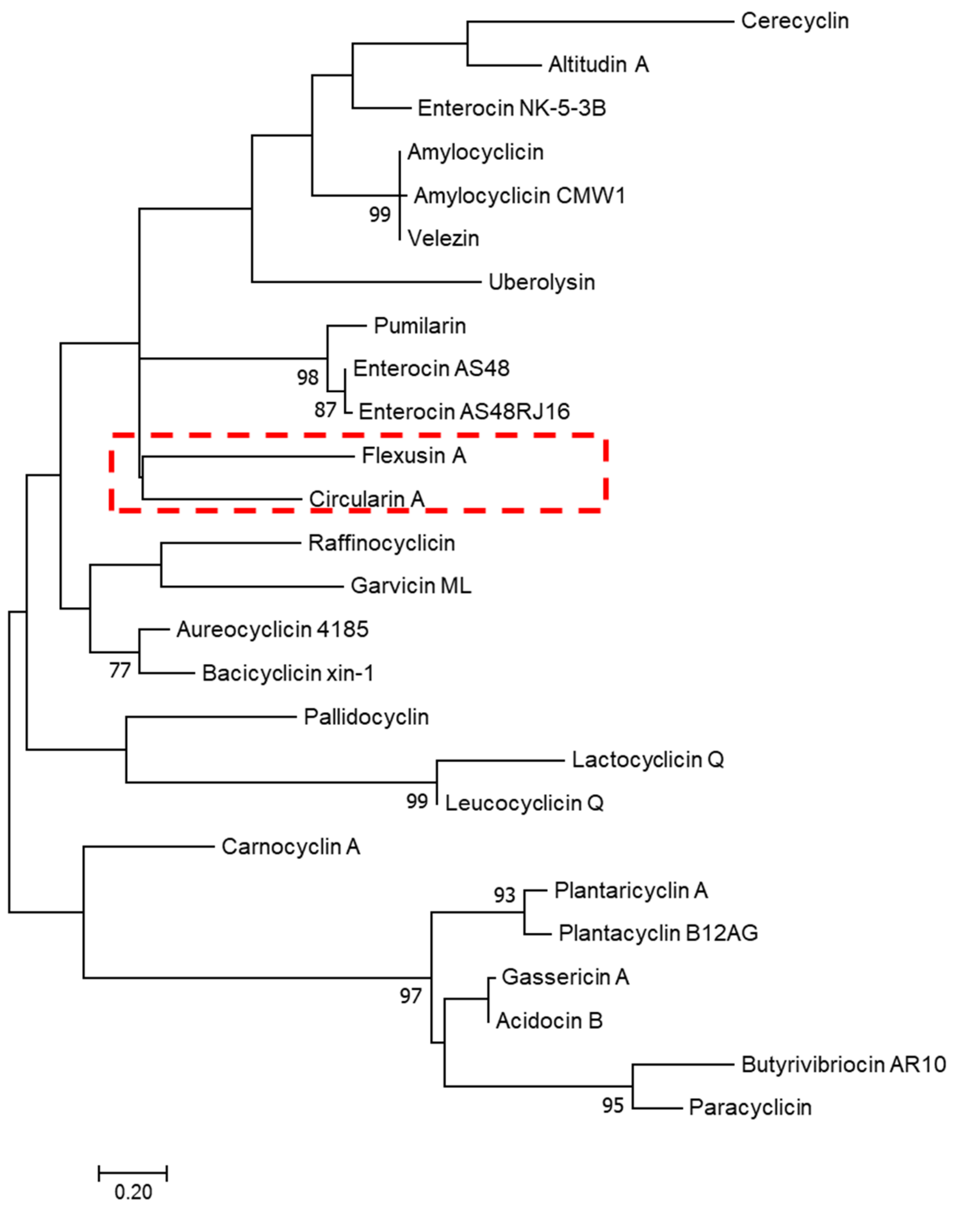

Supplement: Supplementary file 1 [file marinedrugs-23-00095-s001.zip › marinedrugs-3444543-supplementary.pdf]
